# Modified Kirschner wire percutaneous rotation prying reduction combined with elastic stable intramedullary nailing in children with Judet IV radial neck fracture

**DOI:** 10.1186/s12891-023-07008-2

**Published:** 2023-11-13

**Authors:** YongFei Fan, WenQiang Xu, QiXin Liu, ChaoYu Liu, Wei Wang

**Affiliations:** Department of Orthopaedic Surgery, Fuyang people’s Hospital Affiliated to Anhui Medical University, Anhui spinal deformity and clinical medical research center, Fuyang people’s Hospital, Fuyang, 236000 Anhui China

**Keywords:** Percutaneous rotation prying reduction, ESIN, Radial neck fracture, Children

## Abstract

**Purpose:**

This study was to investigate the feasibility and treatment effect of using modified Kirschner wire (K-wire) percutaneous rotation prying reduction combined with Elastic Stable Intramedullary Nailing (ESIN) in children with Judet IV radial neck fracture.

**Methods:**

A retrospective analysis was conducted on 47 children with Judet IV radial neck fracture who underwent treatment with modified K-wire percutaneous rotation prying reduction combined with ESIN from April 2019 to November 2022, including 25 males and 22 females, with an average age of 8.79 years old (ranging from 5 to 14). The study recorded the surgical time, fluoroscopy time, reduction time, and reduction quality evaluated according to the Metaizeau radiological standard. During the last follow-up, the flexion-extension and forearm rotation function of the affected and healthy sides were recorded, and the Mayo Elbow Performance index was used to evaluate the elbow joint function.

**Results:**

The average duration of the operation was 25.51 min (ranging from 14 to 43 min), with a mode of 2 reset times (ranging from 1 to 5) and 8 fluoroscopic times (ranging from 4 to 15). Based on the Metaizeau radiological standard for assessing reduction quality, 45 cases were deemed excellent, while 2 cases were considered good. Following 3–4 weeks of postoperative long-arm cast immobilization, exercises were performed to promote elbow joint and forearm rotation. The ESIN was removed after satisfactory fracture healing around 4 months postoperatively. The average follow-up period was 26.79 months (ranging from 5 to 48). At the final follow-up, the range of motion for the affected limb in flexion, extension, pronation, and supination was (140.23 ± 4.80)°, (4.43 ± 3.98)°, (84.09 ± 4.97)°, and (83.83 ± 4.55)°, respectively. There was no statistically significant difference compared to the healthy side, which had a range of motion of (141.36 ± 3.27)°, (5.28 ± 2.25)°, (85.66 ± 3.20)°, and (84.98 ± 2.57)° (*P* > 0.05). According to the Mayo Elbow Performance index, 44 cases were rated as excellent and 1 case was considered good.

**Conclusion:**

The modified K-wire percutaneous rotation prying reduction combined with ESIN is an effective treatment for severe radial neck fractures in children. This technique offers several advantages, including the ability to easily “capture” significantly displaced radial heads, achieve rapid and accurate reduction, and reduce radiation exposure.

## Introduction

 Radial neck fracture accounts for 5–10% of pediatric elbow fractures. Due to the fact that the epiphysis of the radial neck in children is not yet closed during bone development, the joint capsule and ligaments attached to it have a higher strength than the bone itself, making it more susceptible to fractures from external forces [1, 2]. According to the Judet classification, children with radial neck fractures of type III or higher usually require surgical treatment. Metaizeau proposed the use of percutaneous ESIN fixation for the treatment of such fractures [3–5]. However, for Judet type III and IV radial neck fractures, achieving good reduction with only the rotation technique of the ESIN can be difficult [6].

Many studies have combined percutaneous straight K-wire prying reduction with ESIN rotation reduction to treat severely displaced radial neck fracture, which can improve the quality of reduction [7, 8]. Nevertheless, for severe Judet type IV radial neck fracture, the straight K-wire often has difficulty “capturing” the radial head, and prying reduction requires a large K-wire swing angle, often necessitating multiple attempts. This not only increases surgical trauma and radiation exposure but also prolongs the operation time and may even result in reduction failure and iatrogenic epiphyseal injury, leading to premature epiphyseal closure and radial nerve injury.

Therefore, in order to achieve better reduction and avoid the aforementioned complications, we used the modified K-wire percutaneous rotation prying reduction combined with ESIN to treat children with Judet type IV radial neck fracture. The purpose of this study is to analyze and explore the effectiveness and feasibility of this technology.

## Patients and methods

All patient records at our hospital from April 2019 and November 2022 were retrospectively reviewed. The study was approved by the hospital’s ethics committee, and the guardians of the children authorized the use of their clinical data. The inclusion criteria were as follows: (1) Age ≤ 14 years old; (2) Judet type IV closed radial neck fracture. The exclusion criteria were as follows: (1) Lost to follow-up patients; (2) Patients with open fractures. This study included a total of 47 patients, including 25 males and 22 females, with an average age of 8.79 years old (ranging from 5 to 14). The time from injury to surgery was 1 to 4 days, with an average of 2.02 days. The fracture sites were 20 cases on the left side and 27 cases on the right side. There were 25 cases of isolated radial neck fractures, 19 cases of radial neck fractures combined with proximal ulnar fractures, 2 cases of radial neck fractures combined with humeral medial epicondyle fractures (Table 1). These patients experienced fractures of the proximal ulna and humeral medial epicondyle because of different injury mechanisms. Each patient underwent X-ray and CT examinations before surgery to better observe the displacement direction of the radial neck fracture, so as to facilitate the selection of the K-wire insertion point during surgery and achieve a more satisfactory reduction.

**Table 1:** Patients’ preoperative, intraoperative and postoperative data

	Preoperative	Intraoperative	Postoperative
Age (years)	8.79		
Sex			
Male	25		
Female	22		
Affected side			
Left	19		
Right	28		
Complication			
Proximal ulnar fracture	19		
humeral medial epicondyle fracture	2		
Time from injury to surgery (day)	2.02		
Surgery times (min)		25.51	
Fluoroscopic times		8	
Reset times		2	
Metaizeau radiological evaluation criteria			
Excellent		45	
Good		2	
Fair		0	
Poor		0	
Follow-up time (month)			26.79
MEPI^a^			
Excellent			44
Good			1
Fair			2
Poor			0

^a^*MEPI* Mayo Elbow Performance Index

### Surgical procedure

**Fig. 1:**
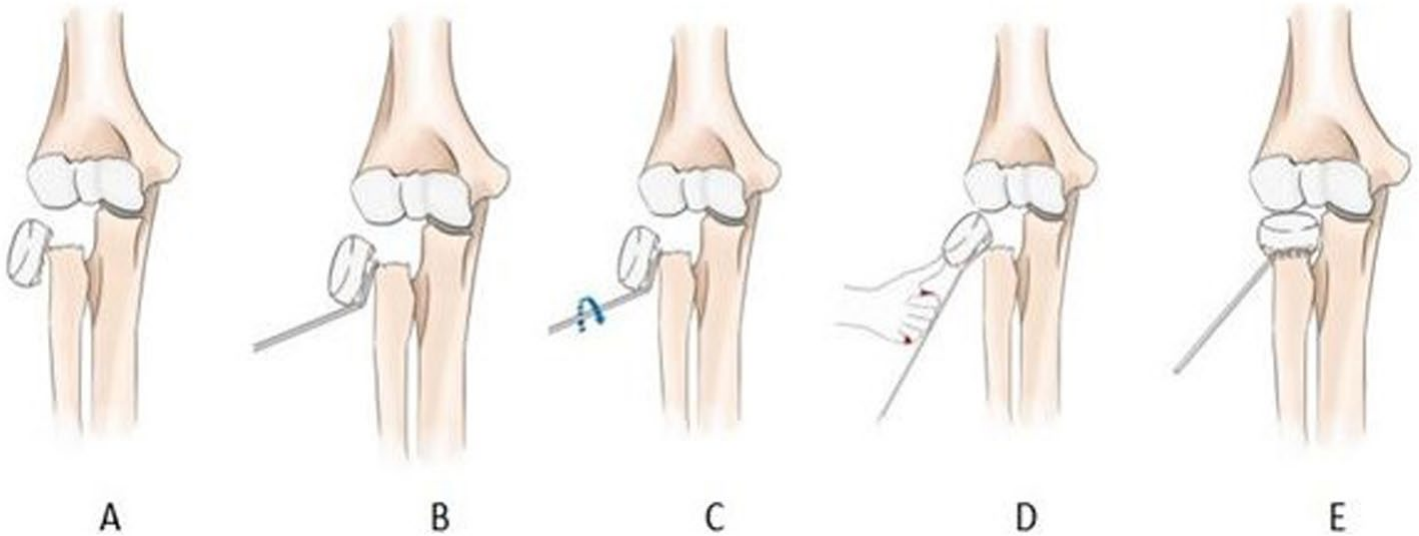
Schematic diagram of modified K-wire percutaneous rotation prying reduction. **A** Judet type IV radial neck fracture; **B** Insertion of pre-bent K-wire; **C** Rotation of K-wire for pry reduction; **D** Assisted reduction with thumb pressure; **E** Satisfactory reduction of the fracture

**Fig. 2:**
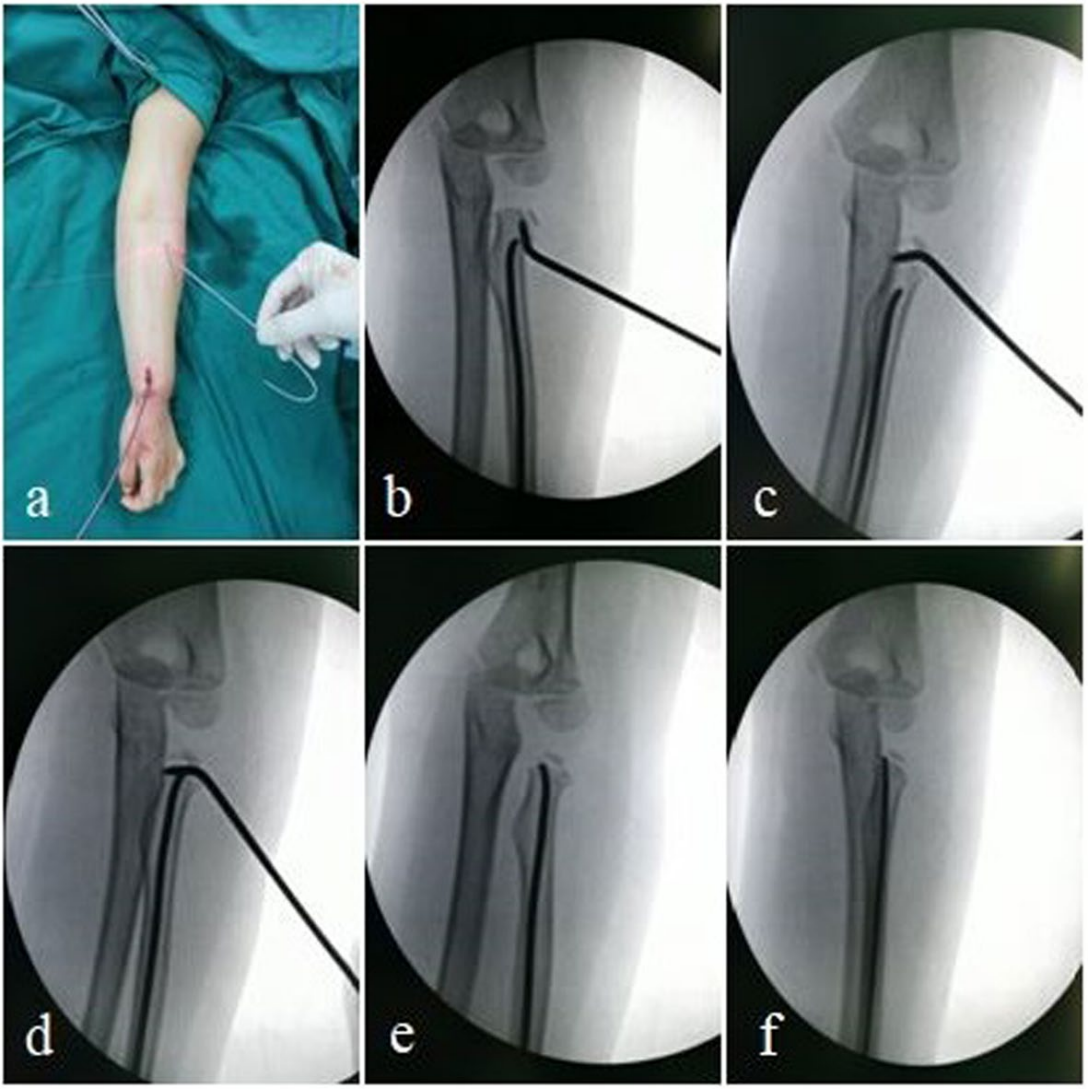
A four-year-old girl presented with left radial neck fracture. **a-f** The reduction steps for radial neck fracture during surgery

All surgery was performed under general anaesthesia and completed by the same surgical team. A 1.5 cm incision was made on the dorsal side of the distal radius, and the entry point for the nail was determined by fluoroscopy at the proximal 1–2 cm of the distal radial epiphysis. An appropriately sized ESIN was inserted into the fracture end 0.5-1 cm. The elbow joint was kept in an extended position, and a K-wire with a diameter of 2.0-2.5 mm was taken, with the tip bent about 45° and a length of 1.5 cm. The tail end was bent in the opposite direction in the same plane, with a length of about 5 cm for gripping. The forearm was pronated appropriately, and the assistant applied traction to the forearm and caused varus of the elbow joint. Under fluoroscopic guidance, the pre-bent K-wire was inserted into the displaced distal radius head through the posterior lateral aspect of the elbow joint until it was inserted between the distal radius head and the radial shaft, capturing the displaced distal radius head. The operator slowly rotated the K-wire inwardly by 180°, using the K-wire to pry the radial neck into reduction, while the operator’s left thumb pressed on the levered distal radius head for reduction. In most cases, a clicking sensation could be felt, and the fracture was reduced under fluoroscopy. The ESIN was then advanced into the radial head. The residual displacement was rotated and adjusted by the curved tail end of the ESIN to achieve accurate reduction and fixation. Finally, the elbow joint was immobilized in a long-arm cast with the elbow flexed at 90° and the forearm in a neutral position (Figs. 1 and 2).

### Follow-up and evaluation

**Fig. 3:**
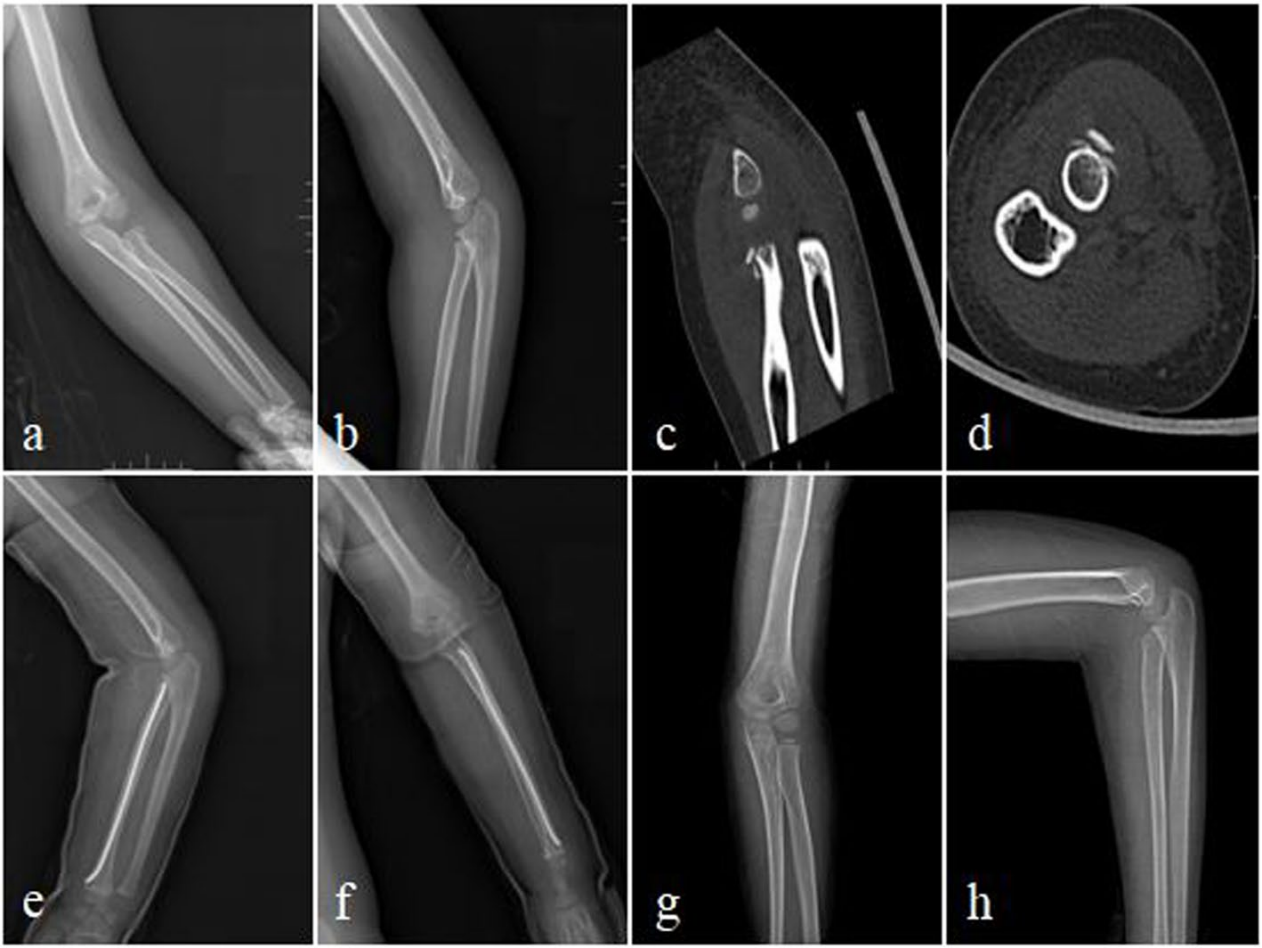
A four-year-old girl presented with left radial neck fracture. **a-d** Preoperative X-ray and 3D CT examination. **e**, **f** X-ray on the first day after surgery. **g**, **h** X-ray at the final follow-up

Three to four weeks after surgery, the cast was removed, and elbow joint flexion and extension, as well as forearm rotation exercises were performed. The surgical time, fluoroscopy times and reduction times were recorded, and the reduction quality was evaluated according to the Metaizeau radiographic criteria. X-ray examinations were performed at follow-up visits, and the ESIN was removed after satisfactory fracture healing at around 4 months postoperatively (Fig. 3). The occurrence of complications such as fracture redisplacement, malunion, radial head necrosis, radial head overgrowth, premature epiphyseal closure, heterotopic ossification, and elbow joint deformity were recorded. At the final follow-up, Mayo Elbow Performance index was used to evaluate the function of the affected limb and compared with the flexion and extension and forearm rotation angles of the contralateral elbow joint.

### Statistical analysis

SPSS 26.0 (version 26.0; IBM, Armonk, NY, USA) was used for statistical analysis. Quantitative data was expressed as means ± standard deviations (SD). The comparison between the affected side and the healthy side was performed using a paired sample t-test. *P* < 0.05 was considered significant.

## Results

All patients underwent modified percutaneous rotation reduction using K-wires, with an average surgical time of 25.51 min (ranging from 14 to 43). The number of rotation reductions during surgery was 2 times (ranging from 1 to 5), and the average number of fluoroscopies was 8 times (ranging from 4 to 15). According to the Metaizeau radiographic criteria, the reduction quality was excellent in 45 cases and good in 2 cases, with an excellent and good rate of 100%. All patients achieved anatomical or near-anatomical reduction, and no iatrogenic radial nerve injury occurred. The accompanying fractures such as the proximal ulna fracture and humeral medial epicondyle fracture, with relatively minor displacement, did not require surgical treatment with internal fixation. After the surgical treatment of the radial neck fracture, they were all immobilized with plaster casts, which did not have a significant impact on the functional recovery of the patient’s elbow joint in the later stages. The follow-up time was 26.79 months (ranging from 5 to 48 months), and according to the Mayo Elbow Performance index at the final follow-up, 44 cases were excellent and 1 case was good (Table 1). There was no statistically significant difference in elbow joint flexion and extension and forearm rotation function between the affected and healthy sides (*P* > 0.05) (Table 2). During follow-up, no complications such as fracture redisplacement, malunion, radial head necrosis, radial head overgrowth, premature epiphyseal closure, heterotopic ossification, or elbow joint deformity occurred in any of the patients.

**Table 2:** Comparison of the angles of flexion, extension, pronation, and supination between the affected and contralateral sides at the last follow-up. (‾*x* ± s)

	Flexion(°)	Extension(°)	Pronation(°)	Supination(°)
Contralateral side	141.36 ± 3.27	5.28 ± 2.25	85.66 ± 3.20	84.98 ± 2.57
Affected side	140.23 ± 4.80	4.43 ± 3.98	84.09 ± 4.97	83.83 ± 4.55
*P* (value)	0.105	0.158	0.120	0.149

## Discussion

The radial neck fracture is a common elbow fracture in children, and different treatment therapies may be adopted depending on the severity of fracture. The mechanism often involves a child falling and the upper limb naturally extending or semi-flexing to support the ground, followed by rotation of the forearm and transmission of force along the forearm. As a result, the radial neck fracture occur when the radial head collides with the small head of the humerus, which is often accompanied by different levels of displacement and recognized as the “tilted hat” sign [9]. The remaining force is then buffered to other parts of the elbow joint, resulting in fractures in corresponding areas. Fractures of this type are commonly classified as Salter-Harris type I/II epiphyseal injuries, and improper treatment may lead to premature epiphysis closure [10].

For patients with Judet type I and II radial neck fractures with an angulation of ≤ 30°, manual reduction and plaster external fixation therapy for 3–4 weeks can be used [6, 11]. Satisfactory results can be achieved by performing elbow and forearm rotation exercises after the removal of external fixation. Shah et al [12, 13] used a modified technique of closed reduction for severely displaced pediatric radial neck fractures, achieving satisfactory results. This technique requires the operator to master special skills of reduction. Due to the poor stability of Judet type III and IV radial neck fractures, surgical treatment is mostly required. The commonly used surgical methods include K-wire-assisted reduction, manual reduction, and Metaizeau method [14–16]. For fractures with significant displacement or unsatisfactory manual reduction, nerve damage is prone to occur during the K-wire levering and reduction process. Metaizeau used ESIN to reduce this type of fracture, which can avoid secondary damage to the epiphysis by not passing through it, reduce the risk of premature epiphysis closure, and allow for earlier removal of plaster external fixation and functional exercise, which is beneficial for the recovery of elbow joint function [17, 18]. However, for Judet type IV fractures with more obvious angulation displacement, the bending angle of the intramedullary nail is limited, and closed reduction by rotation with the ESIN alone often cannot achieve satisfactory reduction [19]. Moreover, relying solely on multiple rotations with the ESIN can easily cause the proximal radial neck to flip and displace, enlarge the medullary cavity, and damage the cancellous bone and epiphyseal plate structure of the neck shaft, leading to loosening of the nail and weakened holding force, which increases the probability of postoperative fracture displacement [20]. Many failed reduction cases require open reduction, which can damage the periosteum and have a negative effect on the blood supply to the radial neck, making it prone to radial head necrosis and premature epiphysis closure in the later stage, as well as causing related complications such as iatrogenic nerve damage, heterotopic ossification, and scar adhesion [4, 21].

The K-wire percutaneous rotation prying reduction combined with ESIN has been widely used in patients with significantly displaced radial neck fractures. This technique uses a straight K-wire inserted percutaneously into the fracture end to directly pry the reduction by using the lateral periosteum “hinge”, which improves the success rate of reduction and achieves satisfactory clinical efficacy [8, 21–23]. However, for Judet type IV radial neck fractures, especially when the angle of the fracture end is close to 90°, the radial head is almost parallel to the radial shaft, and the straight K-wire is hard to insert into the fracture gap. Capturing the radial head with the K-wire is challenging, and increasing the angle of the K-wire leads to excessive prying force during reduction. Moreover, traction and internal rotation of the elbow joint are required to increase the lateral joint gap for reduction, which increases skin tension and makes it difficult to achieve a one-time reduction [10, 24]. In addition, repeated prying with the straight K-wire not only increases radiation exposure but also further damages the bone and epiphyseal plate of the diaphysis, causing iatrogenic epiphyseal plate injury, bone defects, and deep branch of the radial nerve injury [25]. This study modified the straight K-wire lever reduction by pre-bending, which greatly avoids the above drawbacks. The average number of intraoperative fluoroscopies was 8 times, the number of reductions was 2 times, and the average operation time was 25.51 min. According to the Metaizeau radiological evaluation criteria, the excellent reduction rate was 100%, and all cases achieved anatomical reduction or near-anatomical reduction. No complications such as premature epiphyseal closure, radial head necrosis, or deep branch of the radial nerve injury occurred after surgery. At the last follow-up, 44 cases were rated as excellent and one case was rated as good according to the Mayo Elbow Performance index. There was no statistically significant difference in elbow flexion and forearm rotation function compared with the contralateral side.

Advantages of modified percutaneous rotation lever reduction with K-wire and ESIN in the treatment of Judet type IV radial neck fractures in children: (1) The pre-bent tip of the K-wire makes it easy to capture the excessively tilted radial head, avoiding iatrogenic deep branch of the radial nerve and epiphyseal injuries caused by repeated fluoroscopy and puncture adjustment during K-wire lever reduction, and maximizing the protection of the blood supply to the proximal radial neck; (2) The tip of the K-wire is bent at an angle of about 45°, and by rotating 180°, it can easily achieve local lever reduction at a distal angle of 90° or even greater. The main body of the K-wire remains almost in place, making it easy to achieve the “levering angle” that is difficult to complete in one go with a straight K-wire lever reduction; (3) The pre-bent structure of the K-wire tip makes it easy to adjust the direction of the tip, avoiding further damage to the epiphysis caused by the straight K-wire; (4) The ESIN can be used to make minor adjustments to the residual displacement of a few fractures, achieving anatomical or near-anatomical reduction of the fracture.

## Conclusion

To sum up, the modified K-wire percutaneous rotation prying reduction combined with ESIN in the treatment of Judet IV radial neck fracture can achieve satisfactory clinical outcomes. Nonetheless, the sample size incorporated in this analysis is relatively limited and has no control group. Therefore, long-term follow-up with multiple centers and a larger sample size is necessary to validate the findings of the study.

## Data Availability

According to reasonable requirements, the corresponding authors will provide original data to support the conclusions of this paper.
